# Fatty Liver and Fatty Heart—Where do They Stand in the AMIS Syndrome?

**DOI:** 10.3390/healthcare3030666

**Published:** 2015-08-11

**Authors:** W. Wayne Lautt, Zhi Ming, Dallas J. Legare, Kawshik K. Chowdhury, Grant M. Hatch, Hui Helen Wang

**Affiliations:** Department of Pharmacology and Therapeutics, College of Medicine, Faculty of Health Sciences, University of Manitoba, A224-753 McDermot Avenue, Winnipeg, MB R3E 0T6, Canada; E-Mails: wlautt@cc.umanitoba.ca (W.W.L.); zhi-ming@live.com (Z.M.); Dallas.Legare@umanitoba.ca (D.J.L.); kawshik88@yahoo.com (K.K.C.); ghatch@chrim.ca (G.M.H.)

**Keywords:** meal-induced insulin sensitization (MIS), absence of meal-induced insulin sensitization (AMIS), hepatic insulin sensitizing substance (HISS), non-alcoholic fatty liver disease (NAFLD), rapid insulin sensitivity test (RIST), S-adenosylmethionine, vitamins E and C (SAMEC)

## Abstract

Meal-induced insulin sensitization (MIS) refers to the augmented glucose uptake response to insulin following a meal. Absence of MIS (AMIS) causes significant decrease in post-meal glucose disposal leading to postprandial hyperglycemia, hyperinsulinemia, hyperlipidemia, adiposity, increased free radical stress, and a cluster of progressive metabolic, vascular, and cardiac dysfunctions referred to as the AMIS syndrome. We tested the hypothesis that fat accumulation in the liver and heart is part of the AMIS syndrome. Questions examined in the study: (1) Is prediabetic fat accumulation in the heart and liver a component of the AMIS syndrome? (2) Is fatty liver a cause or consequence of peripheral insulin resistance? (3) Is early cardiac dysfunction in the AMIS syndrome attributable to fat accumulation in the heart? and (4) Can the synergistic antioxidant cocktail SAMEC (S-adenosylmethionine, vitamin E, and vitamin C), known to benefit MIS, affect cardiac and hepatic triglyceride levels? Four animal models of AMIS were used in aged male Sprague-Dawley rats (52 weeks ± sucrose ± SAMEC), compared with young controls (nine weeks). Fat accumulation in the heart was not significant and therefore cannot account for the early cardiac dysfunction. Hepatic triglycerides increased only in the most severe AMIS model but the small changes correlated with the much more rapidly developing peripheral adiposity. Systemic adiposity represents an early stage, whereas accumulation of cardiac and hepatic triglycerides represents a late stage of the prediabetic AMIS syndrome. Fat accumulation in the liver is a consequence, not a cause, of AMIS. SAMEC protected against the sucrose effects on whole body adiposity and hepatic lipid accumulation.

## 1. Introduction

Non-alcoholic fatty liver disease (NAFLD) was rarely diagnosed in previous decades, but now has become the most common liver disease in Western countries [1], affecting approximately 30% of the population of the United States [2,3,4]. Progression from fatty liver to steatosis is a later stage, and does not appear to result at HbA1c < 8% [5]. The parallel increase in obesity and diabetes has led to the proposal that accumulated ectopic fat, including that in the liver, leads to peripheral insulin resistance and diabetes [4,6,7]. However, this paradigm does not offer a mechanistic explanation for the cause of elevated hepatic TG, nor for how NAFLD would result in peripheral insulin resistance [8].

We propose a mechanistic paradigm that suggests NAFLD as a component of the AMIS (absence of meal-induced insulin sensitization) syndrome. Meal-induced insulin sensitization (MIS) results through the action of Hepatic Insulin Sensitizing Substance (HISS). HISS doubles the whole-body glucose uptake in response to a bolus of insulin in the fed state, compared to the response in the fasted state [9]. AMIS occurs when HISS action is absent [8].

Following a meal, nutrients are largely partitioned between glycogenic and lipogenic storage. This balance is determined by the proportion of lipogenic and glycogenic insulin action [10] and glycogenic HISS action [8,11,12]. Insulin acts on the liver to cause the release of HISS, but only in the fed state [8,11]. The dynamic biological actions of HISS are demonstrable, and distinct from the direct insulin action on glucose disposal. HISS acts selectively on the skeletal muscle, heart, and kidneys, but not on the liver or adipose tissue [12]. HISS action accounts for approximately 50% of the postprandial glucose disposal response to insulin in rats [13] and two-thirds in humans [14].

The absence of HISS causes a shift in partitioning of storage of nutrient energy from glycogen to fat, leading to an increase in adiposity [15,16]. In conjunction with the metabolic effect, HISS also regulates the postprandial hemodynamic response associated with insulin. The vasodilatory response to insulin during the postprandial state is caused through the action of HISS [17]. Chronic impairment in HISS release causes progression to a cluster of metabolic [15,18], vascular [17], and cardiac [19] dysfunctions. These pathological consequences, secondary to the impairment of HISS release, represent components of what we have called the AMIS syndrome (AMISS) [8,15]. The syndrome progresses from single meal AMIS to adiposity and prediabetes and eventually to type 2 diabetes, where the pancreas can no longer compensate for reduced HISS action.

The AMIS syndrome was studied in animal models of aging [20] and type 2 diabetes [21,22]. Chronic dietary interventions with high-fat diet [22] and 35% sucrose supplement [21] are capable of inducing the early manifestations of AMIS syndrome after as little as two weeks of exposure, by which time HISS action is completely eliminated. AMIS, associated with aging or excess sucrose, results from the impairment of the HISS pathway, while the direct action of insulin remains mostly unaffected at the early stage of AMISS. The progression to the development of AMISS can be prevented and/or reversed by various pharmacological [15,16] and non-pharmacological interventions, including voluntary exercise [23,24,25,26].

It has been suggested that fat accumulation in the heart results in lipid-induced insulin resistance in cardiac myocytes and cardiovascular dysfunction [27], and that fat in the liver causes peripheral insulin resistance [7,28,29,30,31]. Hepatic fat is reported to be strongly associated with fasting plasma insulin concentration [32]. Based on the observation that AMIS results in a shift in nutrient partitioning towards lipids and early and progressive adiposity, we tested the hypothesis that hepatic and cardiac lipid accumulation are also early components of the AMIS syndrome. Does hepatic lipid accumulation lead to reduced HISS-dependent response to insulin, or does the reduced response to insulin precede fatty liver? Can the early cardiovascular dysfunctions associated with the AMIS syndrome be accounted for by cardiac fat accumulation?

For this study we used combinations of animal models that were previously shown to result in manipulation of the AMIS syndrome through affecting the HISS action. Aging is associated with a gradual decrease in MIS [20,33]. A 35% unlimited sucrose liquid supplement results in development of AMIS too rapidly to determine longer term chronology, so the chronic limited volume, low dose 5% sucrose supplement model was used [20]. A synergistic antioxidant cocktail (SAMEC: S-adenosylmethionine, vitamin E, and vitamin C) was developed as a research tool and has been shown to provide protection against the development of AMIS syndrome through preservation of HISS action (reviewed by Wang *et al.*, [16]). The current models, which have varying degrees of AMIS, were combined in order to determine the sequence of appearance and the correlation of hepatic and cardiac TG with other signs and symptoms of the AMIS syndrome.

The results show that fat accumulation in the liver is a very late stage of prediabetes and is not a cause, but a consequence, of the AMIS syndrome. Cardiac TG remained insignificantly altered, but the small changes pooled for all groups showed correlation with AMIS. Cardiac dysfunction has previously been shown to be a much earlier consequence in the same animal models [19]. Cardiac dysfunctions occur early in the AMIS syndrome and well prior to significant cardiac lipid accumulation. Cardiac and hepatic lipid accumulations occur later and develop at a slow rate, but in correlation with other AMIS syndrome parameters (e.g., whole-body and regional adiposity).

## 2. Experimental

**Figure 1:**
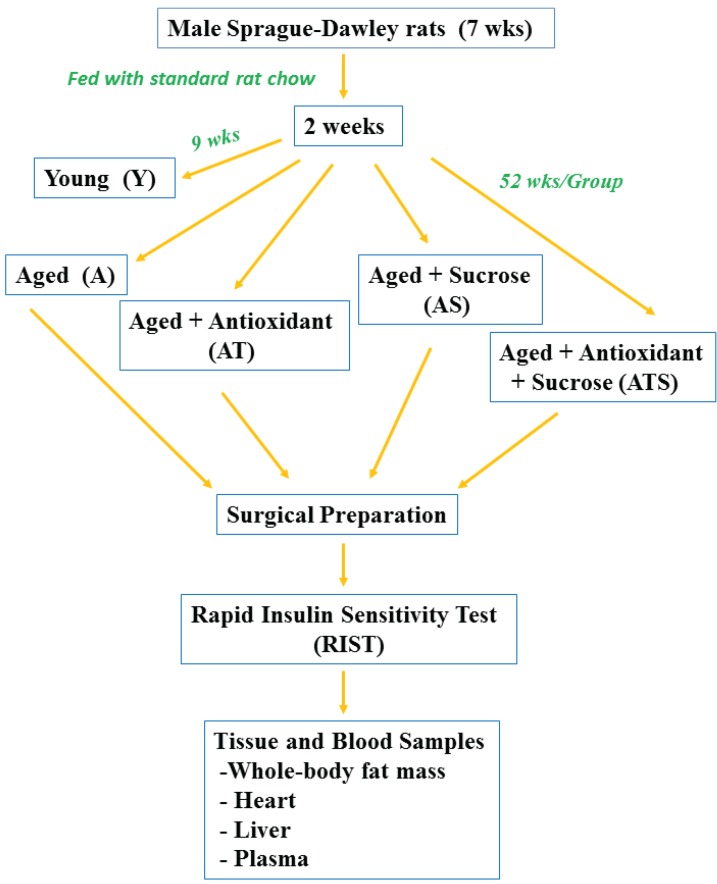
The methodology flow chart for testing animals. wks = weeks of age.

The flow chart of methodologies used in the testing of animals in this study is shown in Figure 1.

### 2.1. Animals and Groups (n = 12–18/group)

Animals were treated according to the guidelines of the Canadian Council on Animal Care. The Protocol Management and Review Committee at the University of Manitoba approved all protocols.

Male Sprague-Dawley rats (Charles River, St. Constant, QC, Canada) of seven weeks old (body weight 200–225 g) were pair-housed and maintained under controlled conditions (22 ± 1 °C, 12 h light/12 h dark cycle). They were fed a standard rat chow diet (caloric intake: 60% carbohydrates in corn starch, 25% protein, and 14% lipids [19]) with free access to tap water for two weeks to adapt to the housing environment. Then the animals were divided into four groups to be maintained until 52 weeks of age. (1) Aged (A): maintained on normal chow and regular tap water only; (2) Aged and antioxidant treatment (AT): maintained on normal chow supplemented with the antioxidant cocktail SAMEC (S-adenosylmethionine (SAM, 0.5 g/kg diet), vitamin E (1500 IU/kg diet), and vitamin C (12.5 g/kg diet)) and regular tap water; (3) Aged with sucrose supplementation (AS): maintained on normal chow, drinking water containing 5% sucrose (50 mL/rat/day) plus access to regular tap water; and (4) Aged and antioxidant treatment with sucrose supplementation (ATS): maintained on normal chow supplemented with SAMEC, drinking water containing 5% sucrose water (50 mL/rat/day) plus access to regular tap water. Given the average daily food consumption of 20 g and the average body weight of 0.8 kg, the approximate daily intake for SAM was 12.5 mg/kg body weight, vitamin E: 37.5 IU/kg body weight and vitamin C: 312.5 mg/kg body weight. Rats in these four groups were tested at 12 months of age. Young adult group (Y) (nine-week) was maintained on standard rat chow and regular tap water. Y-group served as the young control for 12-month-old groups. These 52-week-olds were “aged” rats although they were not at the end of their life span. Body weight gain was monitored once every two weeks. Food and water intake were monitored for one-week periods throughout the treatment. The animal identification was assured by microchip implantation.

### 2.2. Surgical Preparation

To establish a consistent postprandial state, all rats underwent an 8 h fast and a re-feeding period of 2 h immediately before the start of surgical preparation. As described previously [18,19,34], the rats were anesthetized with an intraperitoneal injection of sodium pentobarbital (54.7 mg/kg; CEVA Sante Animal S.A., Libourne, France). Anesthesia was maintained by a continuous infusion of sodium pentobarbital (0.5 mg·mL^−1^ saline given at 50 µL·min^−1^) through a cannula in the jugular vein, supplemented with a 0.54 mg (0.01 mL) bolus injection when required. The rats were placed on a temperature-controlled surgical table (Harvard Apparatus, Kent, England) and rectal temperature was monitored and held at 37.0–37.5 °C. Spontaneous respiration was allowed through a tracheal catheter.

An arterial-venous shunt was established, as described [34], for monitoring the mean arterial blood pressure (MAP), derivation of arterial blood samples, and intravenous drug delivery. Briefly, two catheters (polyethylene tubing PE60), one inserted into the right femoral artery and the other into the right femoral vein, were connected with silicon tubing. A side branch of the circuit was connected to a pressure transducer for recording of the shunt pressure which, when silicon tubing on the venous side of the circuit was clamped, measured the systemic arterial blood pressure. Blood samples were taken from the arterial side of the shunt for glucose measurement. Flowing blood within the shunt assures real-time measurement of the arterial blood glucose concentration, which is essential for the dynamic euglycemic clamp test described below. An infusion line was inserted into the venous side of the shunt for intravenous drug delivery. Another infusion line connected to the jugular vein was established for glucose infusion. Animals were heparinized (100 IU·kg^−1^) to prevent blood clotting in the vascular shunt.

### 2.3. Rapid Insulin Sensitivity Test (RIST)

The RIST was performed as previously described [34]. Briefly, following completion of surgery, animals were allowed a 30 min stabilization period. The baseline glucose level was then determined from samples taken at 5-min intervals and continued until three successive stable determinations were made. The mean value of these three glucose concentrations was used as the baseline for the RIST. A blood sample was taken for determination of insulin, plasma enzymes, and lipids. To perform the RIST, human insulin (50 mU·kg^−1^ in 0.5 mL saline, Novolin^®^ge Toronto, Novo Nordisk, Bagsvaerd, Denmark) was infused intravenously via the shunt at the rate of 0.1 mL·min^−1^ for 5 min. After 1 min of insulin infusion, the first test glucose sample was taken and a variable glucose infusion (10%) was initiated. Blood samples were taken every 2 min and the glucose infusion rate was adjusted to maintain euglycemia in the animal. The RIST index was the amount of glucose (mg·kg^−1^) infused to maintain euglycemia over the test period, which was terminated when no further glucose infusion was required (approximately 35 min). At the end of a RIST, the animal normally returned to its pretest glycemic level (± 5%). A data acquisition system (National Instruments Lab-View, Austin, TX, USA) combined with application software was used to record and analyze the mean arterial blood pressure, to calculate the RIST index, and to provide real-time monitoring of adherence to the euglycemic baseline. The software program calculated accuracy and precision for the maintenance of the euglycemic target baseline. If either deviated by more than 5%, the entire RIST was considered to be invalid and discarded. Blood glucose concentration was measured using the glucose oxidase method (Yellow Springs Instruments, Yellow Springs, OH, USA).

Two RISTs were performed for each rat. Following establishment of the glucose baseline, the first RIST index was determined and used as the control (fed) RIST. The second RIST was performed after blockade of HISS release with intravenous infusion of atropine (1 mg·kg^−1^ in 0.5 mL saline, 0.1 mL·min^−1^) [13,34] and reestablishment of a stable glycemic baseline. The use of atropine in this regard has been reviewed [35]. The first RIST index includes the effects of both HISS-dependent and HISS-independent components, and the second RIST index represents only the HISS-independent component of insulin action [34]. The percentage contribution of HISS-dependent component (HISS action) to total insulin action was calculated as: %HISS = [(RIST index in control – RIST index after atropine)/RIST index in control] × 100%. The percentage contribution of HISS-independent component (direct insulin action) to total insulin action was calculated as: %Insulin = RIST index after atropine/RIST index in control × 100%.

### 2.4. Tissue and Blood Samples

Whole-body fat mass (FM) was estimated using electrical impedance measurement [8]. Fat mass % was calculated as the ratio of fat to body weight × 100%. Fat tissue from the epidydimal, perinephric, and perienteric fat pads was collected and weighed. HOMA-IR (homeostatic model assessment of insulin resistance) was calculated from the fasting insulin and glucose levels (Fasting insulin × Fasting glucose/22.5).

Prior to termination of the experiment, the heart and right lobe of the liver were harvested and quickly washed with ice-cold saline to remove blood from the tissue surface. A 5 × 5 mm sample of tissue from the liver and 3 × 3 mm samples of tissue from both the left and right ventricles of the heart were collected and flash frozen with dry ice. The tissues were maintained at −80 °C until the time of tissue analysis, during which triglycerides (TG) were isolated and quantified as described [36]. In some experiments, tissue total fatty acid-containing TG was quantified as described [37].

Plasma levels of non-esterified fatty acids (NEFA) were quantified using a commercial kit (HR Series NEFA-H, Wako Diagnostics, USA). 20 μL of plasma was placed in a small glass tube, and 200 μL of color regent solution A was added to each tube. The samples were mixed well and placed in a water bath incubator for 10 min at 37 °C. 100 μL of color reagent solution B was then added and the suspension mixed well and incubated for a further 10 min at 37 °C. Absorbance was measured at 550 nm, and NEFA levels were determined based on a standard curve with known NEFA concentrations. All samples were assayed in triplicate.

### 2.5. Chemicals

Human insulin was purchased from Novo Nordisk Canada Inc. (Mississauga, ON, Canada). Atropine, Vitamin C (L-Ascorbic acid), and Vitamin E ((±)-α-Tocopherol) were all purchased from Sigma-Aldrich (Oakville, ON, Canada). S-adenosylmethionine was purchased from Now Foods (Bloomingdale, IL, USA). Insulin and atropine were dissolved in saline. The antioxidants were incorporated into regular rat chow by Research Diets, Inc. (New Brunswick, NJ, USA). Plasma insulin concentration was assayed by ELISA (ALPCO, Windham, NH, USA). Alanine transaminase test kit was from Cayman Chemical Co. (Ann Arbor, MI, USA). An aspartate aminotransferase test kit was obtained from BioVision Inc. (Milpitas, CA, USA).

### 2.6. Statistical Analysis

A one-way ANOVA followed by Tukey’s test was employed when the multiple means from different groups were compared. Statistical significance was considered at p < 0.05. Linear regression analysis was performed to explore the correlations of any two variables. GraphPad Prism 5.0 (La Jolla, CA, USA) was used as the statistical software to perform the analysis.

## 3. Results and Discussion

### 3.1. Insulin and HISS Action

**Figure 2:**
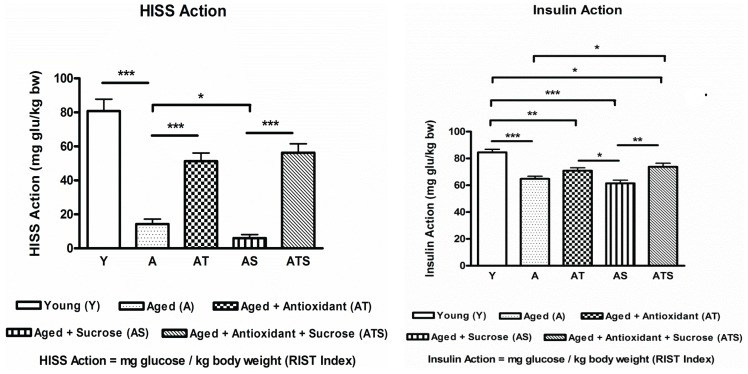
The effects of age ± sucrose supplementation and SAMEC protection on HISS and insulin action. HISS and insulin action determined from the RIST in young (Y), 52-week-old or aged (A) rats, aged rats supplemented with sucrose (AS), and ± SAMEC protection. Aging to one year decreased both HISS action and insulin action. Sucrose supplementation further aggravated the conditions in aged rats. The proportion of glucose disposal attributed to insulin increased to 91% of the total, as HISS action declined in the AS group. The SAMEC treatment (ATS) conferred protection of HISS action. * *p* < 0.05; ** *p* < 0.01; *** *p* < 0.001.

The study was designed to further characterize the AMIS syndrome and determine if fatty liver and heart are components of the AMIS syndrome. Aging was associated with a reduction in total glucose uptake and HISS action. A low-dose sucrose supplement provided during the aging process resulted in a further reduction (Figure 2). The young group (Y) had a RIST index of 165.3 ± 8.2 mg/kg, and of that HISS action accounted for 50.0% ± 2.0% (*i.e.*, 80.8 ± 7.0 mg/kg) of total glucose uptake. At 52 weeks of age (A), total glucose uptake decreased to 79.1 ± 4.0 mg/kg, and HISS action accounted for only 17.0% ± 2.7% (*i.e.*, 14.3 ± 3.0 mg/kg) of glucose uptake. The sucrose supplement in the aged group (AS) resulted in a further significant decrease in total glucose uptake to 67.5 ± 3.6 mg/kg and HISS action to 7.8% ± 2.2% (*i.e.*, 6.0 ± 2.1 mg/kg). SAMEC incorporated into the diet protected against the decrease in HISS action that occurs with aging (AT: total glucose uptake = 130.1 ± 7.51 mg/kg; HISS-dependent glucose uptake = 56.3 ± 5.22 mg/kg), and especially protected against the negative metabolic effects of the sucrose-supplemented diet (ATS: total glucose uptake = 130.1 ± 7.5 mg/kg; HISS-dependent glucose uptake = 56.3 ± 5.2 mg/kg) (Figure 2, Table 1).

### 3.2. Fasting and Postprandial Metabolic Parameters

Fasting glucose was elevated with age and was protected (49%) by SAMEC. SAMEC also resulted in a significant reduction in fasting glucose in the old rats on sucrose (Table 1). Age resulted in a significant elevation in 2 h postprandial blood glucose, which was further elevated by sucrose. SAMEC had statistically non-significant protection (31%) against the effect of aging on 2 h postprandial blood glucose. However, SAMEC conferred a complete protection against the additional impact of sucrose in the aged (ATS) group (Table 1).

**Figure 3:**
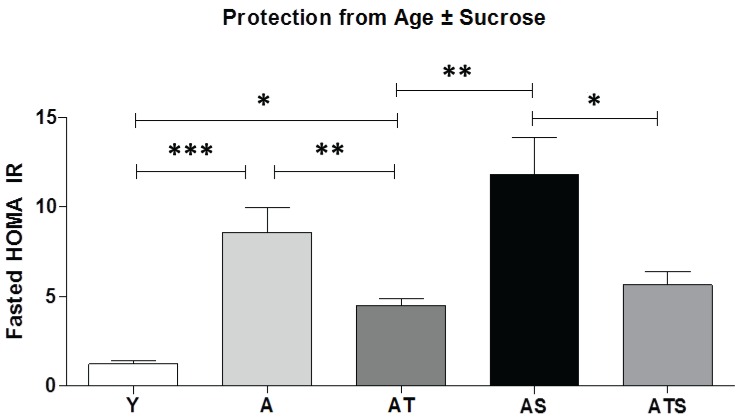
SAMEC protection from the effects of age ± sucrose supplementation on the homeostatic model assessment (HOMA). The HOMA-IR data showed protection by SAMEC in aged ± sucrose-supplemented groups. * *p* < 0.05; ** *p* < 0.01; *** *p* < 0.001.

Plasma insulin levels in the fasted and fed states were significantly elevated with age, and SAMEC provided protection (Table 1). The sucrose diet raised the postprandial insulin levels over eight-fold compared to the young group and almost double the level produced by age alone. SAMEC conferred complete protection (134%) against the effect of sucrose in the aged rats (Table 1). Homeostatic model assessment (HOMA) is a method used to quantify insulin resistance estimated from fasting glucose and insulin levels. Calculated HOMA-IR values decreased in AT and ATS groups, indicating preservation of insulin sensitivity with SAMEC supplementation in the aged ± sucrose groups (Figure 3).

**Table 1:** Physical and metabolic parameters in young and aged ± sucrose-supplemented and SAMEC-treated groups.

	Nine-Week-Old Rats	52-Week-Old Rats				
Parameters	Young Controls (Y)	Aged (A)	Aged Sucrose (AS)	Aged Treated (AT)	Aged Treated Sucrose (ATS)	*p < 0.05*
Body Weight g	366 ± 112.8 (12)	795 ± 24.1 * (12)	853 ± 26.6 * (12)	783 ± 21.4 * (12)	802 ± 19.4 * (12)	* vsY
MAP mmHg	114 ± 3.7(12)	121 ± 3.7 (12)	114 ± 4.3 (12)	114 ± 5.2 (12)	118 ± 5.0 (12)	*Ns*
Fasted Glucose mg/dl	80.1 ± 3.0 (11)	99.0 ± 2.6 * (12)	102.8 ± 3.2 * (12)	89.7 ± 1.7 (12)	91.3 ± 2.4 *§ (11)	* vsY, § *vsAS*
Fed Glucose mg/dl	105.4 ± 5.0 (11)	122.5 ± 4.7 (12)	147.3 ± 11.6 * (12)	117.2 ± 5.0 (11)	121.6 ± 5.0 § (11)	* vsY, § *vsAS*
Fasted Insulin μg/L	0.307 ± 0.04 (11)	1.65 ± 0.26 * (11)	1.72 ± 0.28 * (11)	0.90 ± 0.07 (11)	1.17 ± 0.16 * (11)	* vsY, § *vsAS*
Fed Insulin μg/L	2.63 ± 0.42 (11)	9.79 ± 2.34 § (11)	17.22 ± 3.13 * (11)	7.12 ± 0.91 (11)	7.26 ± 0.85 § (11)	* vsY, § *vsAS*
Whole Body FM g	31.9 ± 2.1 (14)	263.6 ± 13.7 *§ (12)	331.6 ± 17.3 * (13)	221.5 ± 17.0 * (14)	249.0 ± 13.1 *§ (13)	* vsY, § *vsAS*
Whole Body FM %	8.7 ± 0.4 (14)	33.4 ± 1.2 *€ (12)	38.2 ± 1.0 * (13)	28.4 ± 1.6 * (14)	30.8 ± 1.4 *§ (13)	* vsY, § *vsAS* € *vsAT*
Total Visceral FM g	11.6 ± 0.8 (14)	83.4 ± 3.9 *§ (12)	105.8 ± 6.1 * (13)	69.3 ± 5.6 * (14)	80.8 ± 4.9 *§ (13)	* vsY, § *vsAS*
Total Visceral FM %	3.18 ± 0.17 (14)	10.6 ± 0.3 *€ (12)	12.2 ± 0.38 * (13)	8.9 ± 0.57 * (14)	10.0 ± 0.54 *§ (13)	* vsY, § *vsAS* € *vsAT*
Plasma Triglycerides mmol/L	0.57 ± 0.11 (14)	1.76 ± 0.24 *§ (11)	3.24 ± 0.40 * (12)	1.81 ± 0.19 * (12)	1.90 ± 0.25 *§ (12)	* vsY, § *vsAS*
Total Chol/HDL Chol	1.26 ± 0.03 (14)	1.69 ± 0.12 * (12)	1.80 ± 0.11 * (11)	1.65 ± 0.09 * (12)	1.62 ± 0.06 *§ (13)	* vsY, § *vsAS*
AST	212.3 ± 13.1 (14)	316.7 ± 48.9 (12)	309.0 ± 31.0 (14)	293.4 ± 26.7 (11)	295.4 ± 23.8 (14)	*Ns*
ALT	69.5 ± 4.7 (14)	117.3 ± 13.8* (12)	121.6 ± 14.0 * (14)	117.5 ± 14.1 * (11)	107.3 ± 10.7 (14)	* vsY

### 3.3. Hepatic and Cardiac Triglycerides (TG), and Body Fat

**Figure 4:**
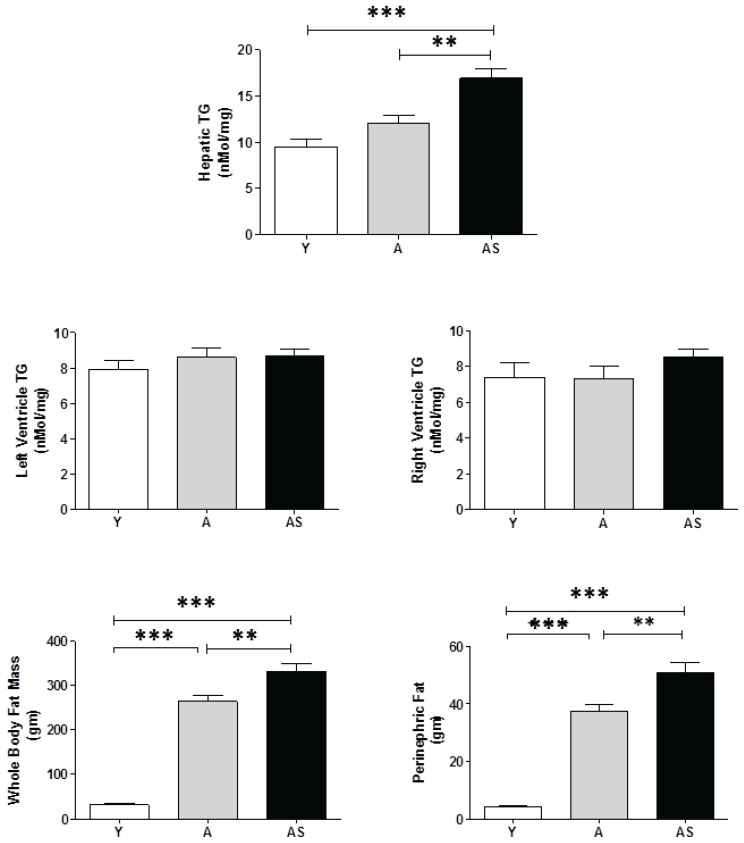
The effects of age ± sucrose supplementation on hepatic and cardiac triglycerides, and body adiposity. Hepatic TG was not significantly increased with aging alone, but was with sucrose supplementation. Cardiac TG in the left and right ventricles did not change when comparing rats that were young (Y), aged (A), and aged on sucrose (AS). However, perinephric fat increased with aging ± sucrose supplementation, as did whole-body fat mass. gm = g. * *p* < 0.05; ** *p* < 0.01; *** *p* < 0.001.

Hepatic TG was not significantly different between young and aged rats. However, sucrose resulted in a significant increase in hepatic TG in the AS group (Figure 4). TG in the heart was not elevated by age or sucrose in either the left or right ventricle (Figure 4). The mean values were within a tight range (7.4–8.7 nmol/mg) for all five groups. SAMEC had no effect on cardiac TG in aged ± sucrose groups. In addition, SAMEC did not have effect on hepatic TG in the aging group (A *vs.* AT: 12.1 ± 0.8 *vs.* 12.7 ± 0.8 nmol/mg); however, it prevented the sucrose effect on liver fat in the aged + sucrose group (AS *vs.* ATS: 17.0 ± 1.0 *vs.* 14.2 ± 0.5 nmol/mg). Impaired liver function, as manifested by elevation of the liver enzymes (AST and ALT), was found in aged ± sucrose groups. SAMEC had no effects on the liver enzymes (Table 1).

**Figure 5:**
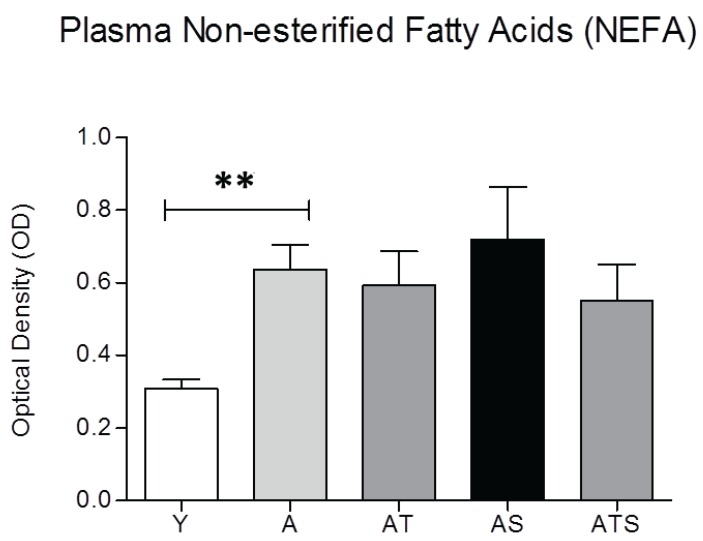
Plasma non-esterified fatty acid (NEFA) levels in different study groups. Plasma NEFA concentrations were significantly increased with all aged groups, but remained unaffected by sucrose ± SAMEC. ** *p* < 0.01.

Plasma TG levels were increased by age and further doubled by sucrose. The sucrose effect was prevented by SAMEC (90%), but the effect on age-related elevation in TG was not significant (Table 1). The beneficial effect of SAMEC on the ratio of total to HDL cholesterol was non-significant in the AT group, but was significant in the sucrose (ATS) group (Table 1). An increase in the ratio of total cholesterol to HDL cholesterol is a predictor of the rise in cardiometabolic risks. Total cholesterol *vs.* HDL cholesterol increased with age and was further aggravated by sucrose supplementation, which was protected by SAMEC (Table 1). The plasma non-esterified fatty acid (NEFA, free fatty acids) levels increased with age but remained unaffected by sucrose ± SAMEC in aged groups, although the trends follow the pattern of AMIS symptoms and may be related to the small but significant changes in direct insulin action (Figure 5).

The whole-body FM and FM% were elevated with age ± sucrose (Figure 4, Table 1), and decreased significantly with SAMEC supplementation (Table 1). The fat pad mass (sum of perinephric, epidydimal, and perienteric fat pads) and fat pad mass % increased significantly with age ± sucrose (Figure 4, Table 1), and was significantly prevented by SAMEC (Table 1).

**Figure 6:**
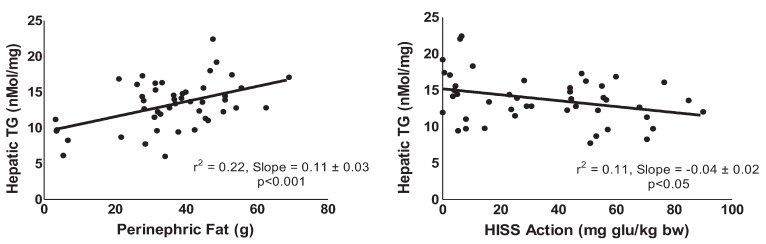
Correlation of hepatic triglycerides with fat pad mass and HISS action. Although total body and regional fat pad masses increase early in AMIS syndrome, hepatic TG elevations become significant only in the aged ± sucrose groups. Also, a significant correlation (r^2^ = 0.22; *p* < 0.001) between hepatic TG and perinephric fat indicates that the fatty liver is a later, but predictable, component of the AMIS syndrome. An inverse correlation (r^2^ = 0.11; *p* < 0.05) exists between HISS action and hepatic TG.

The fat pad mass at each of the anatomical sites correlated with the small changes in total hepatic TG (e.g., hepatic TG *vs.* perinephric fat: *p* < 0.001, r^2^ = 0.22, Figure 6). Fasting or fed glucose showed no significant correlation with hepatic TG. Fasting (*p* < 0.001, r^2^ = 0.27) and postprandial (*p* < 0.03, r^2^ = 0.1) insulin was correlated with hepatic TG. There was also a negative correlation between hepatic TG and HISS action (*p* < 0.05, r^2^ = 0.12, Figure 6).

### 3.4. AMIS, Hepatic and Plasma Triglycerides and NEFA

Aging resulted in a statistically significant decrease in HISS action and an increase in adiposity, and a small but not significant elevation in hepatic TG that was potentiated by the sucrose supplementation (Figure 4). The sucrose supplement to the aging animals had a strong impact on AMIS and components of the AMIS syndrome (Table 1) that correlated with the small and late changes in hepatic TG levels (Figure 6). The effect of the sucrose supplement on hepatic TG was completely prevented by SAMEC. Whether the effect of SAMEC on hepatic TG is acting entirely through protection of HISS action is not clear. The correlation of HISS action with hepatic TG and that with body adiposity suggests that the SAMEC benefits may be primarily through protection of HISS action.

Our data are inconsistent with the suggestion that fat accumulation in the liver may be the primary event leading to peripheral insulin resistance [7,29,30]. The aging models of AMIS did not show significant elevation in hepatic TG but did show peripheral adiposity and reduced response to insulin (HISS-dependent). The rate of development of peripheral adiposity was ~10 times the rate of hepatic TG accumulation (Figure 6). However, in models of more advanced AMIS with age + sucrose, the liver fat content was significantly increased. Hepatosteatosis involves inflammatory responses as well as accumulation of hepatic TG. By the time subjects are diagnosed with diabetes, 68% have been reported to have hepatosteatosis. Fatty liver was associated with higher body mass index, diastolic blood pressure, mean blood pressure, TG, and lower HDL levels [38].

In contrast to the hepatic TG (which was increased only in the advanced AMIS model of aging + sucrose), the plasma TG was significantly increased in the aged group, and was further doubled by sucrose (Table 1). The increase in plasma TG, along with the increased whole body and visceral adiposity, is an early pathology of AMIS resulting from a shift in nutrient partitioning from glycogen to fat. The sucrose effect on plasma TG was prevented by SAMEC, but the effect on age-related elevation in TG was not significant (Table 1).

The plasma non-esterified fatty acid (NEFA) levels increased with age, but remained statistically unaffected by sucrose ± SAMEC treatment in aged rats (Figure 5), although NEFA tended to change with the degree of HISS action from the various groups. Therefore, we cannot pinpoint the exact role of altered NEFA metabolism, as reflected by plasma levels, or label it as a component of the AMIS syndrome.

Macedo’s group showed that the heart is a site of HISS action [12]. Although cardiac and renal glucose uptake accounted for less than 7% of the total glucose disposal, HISS action accounted for 35% of the cardiac glucose uptake in response to insulin.

Cardiac TG was not significantly altered in the left or right ventricle in any of the groups, including the age + sucrose group. This observation clearly dissociates cardiac dysfunction from cardiac TG accumulation. Similar groups of rats had cardiac dysfunction assessed from the left ventricular pressure-volume loops [19]. Cardiac dysfunctions (e.g., reduction in the cardiac index, heart rate, ejection fraction, and increase in left ventricular end-diastolic pressure and total peripheral vascular resistance) also increased with age. The impaired cardiac performance was significant at 26 weeks and had progressed by 52 weeks of age, which was shown to be strongly correlated with the degree of AMIS, and strongly protected by SAMEC [19]. These progressive dysfunctions occurred in the absence of significant alterations in cardiac TG in either the left or right ventricle (Figure 4). Although these previous studies indicate cardiac dysfunction as being a much earlier component of the AMIS syndrome, occurring in parallel with whole-body adiposity, the current study shows that fat accumulation in the heart does not account for this early cardiac dysfunction.

## 4. Current Status of the AMIS Syndrome Hypothesis

An objective of this study was to extend the recent knowledge related to the progressive AMIS (absence of meal-induced insulin sensitization) syndrome to determine: if fat accumulation in the liver and heart are components of the AMIS syndrome; if fatty liver is a cause or consequence of peripheral HISS-dependent insulin resistance; if fat accumulation in the heart can be attributed with early cardiac dysfunction in the AMIS syndrome; if a preventative of the AMIS syndrome can affect hepatic and cardiac TG levels.

Based upon our series of studies in the past decade, the AMIS syndrome has been defined as a predictable progression of dysfunctions that are initiated by a chronic alteration in nutrient partitioning regulated by HISS and insulin. AMIS begins with failure of HISS secretion in response to a meal. Normally, in the presence of two synergistic permissive feeding signals (activation of hepatic parasympathetic nerves and elevation of hepatic glutathione level) [11,39], insulin causes the hepatic release of HISS [40]. HISS action doubles the glucose disposal response to insulin in the postprandial state as a result of selective action in the skeletal muscle, kidneys, and heart [12]. HISS action is additive to insulin action for the hypoglycemic effect. However, HISS is glycogenic and insulin-sparing, whereas insulin has strong lipogenic actions. Nutrient energy is primarily partitioned to skeletal muscle by HISS, and to the liver and adipose tissue by insulin.

AMIS results when HISS is not released following a meal. The absence of HISS action for one meal has immediate metabolic [9] and vascular [19] consequences. The metabolic consequences of impaired HISS action are postprandial hyperglycemia and compensatory hyperinsulinemia, which lead to nutrient storage primarily as lipid rather than glycogen [20,41]. The progression of the signs and symptoms of the AMIS syndrome depends upon the degree and duration of AMIS. In order to determine a sequence of development of signs and symptoms attributable to AMIS, the present study used four models of varying degrees of AMIS: aged (52 weeks) ± sucrose supplement, and the effect of SAMEC on both conditions. This approach has previously been a useful tool to test the AMIS syndrome hypothesis [20]. Elevated fasting and postprandial insulin levels and accumulated adiposity are among the earliest chronic signs. Increasing the degree of AMIS by a sucrose supplement or decreasing AMIS through the use of SAMEC resulted in quantifiable changes in the degree of AMIS, which correlated strongly with whole body and fat pad content (confirmed in the present study, Table 1).

## 5. Conclusions

The AMIS syndrome is associated with the early lipid dysfunction that is seen as increased plasma lipids, whole-body adiposity, and mass of fat pads at an early stage. Cardiac dysfunction, related to the AMIS syndrome, occurs in the absence of elevated TG levels in either the left or right ventricle. If elevated cardiac TG level is a component of the AMIS syndrome, it is at a much later stage, well after generalized adiposity and after elevated hepatic TG. Elevated hepatic TG was seen only in the most severe stage of these models. Elevated hepatic TG is clearly not the cause of peripheral insulin resistance and the AMIS syndrome, but rather a late stage consequence of the prediabetic phase of the AMIS syndrome. SAMEC was confirmed to confer significant protection against signs and symptoms of the AMIS syndrome, including preventing the elevation in hepatic TG caused by sucrose supplementation in aged rats.

## References

[1] Erickson S.K. (2009). Nonalcoholic fatty liver disease. J. Lipid Res..

[2] Browning J.D., Szczepaniak L.S., Dobbins R., Nuremberg P., Horton J.D., Cohen J.C., Grundy S.M., Hobbs H.H. (2004). Prevalence of hepatic steatosis in an urban population in the United States: Impact of ethnicity. Hepatology.

[3] Farrell G.C., Larter C.Z. (2006). Nonalcoholic fatty liver disease: From steatosis to cirrhosis. Hepatology.

[4] Catta-Preta M., Mendonca L.S., Fraulob-Aquino J., Aguila M.B., Mandarim-de-Lacerda C.A. (2011). A critical analysis of three quantitative methods of assessment of hepatic steatosis in liver biopsies. Virchows Arch..

[5] Christman A.L., Lazo M., Clark J.M., Selvin E. (2011). Low glycated hemoglobin and liver disease in the U.S. population. Diabetes Care.

[6] Magkos F. (2012). Putative factors that may modulate the effect of exercise on liver fat: Insights from animal studies. J. Nutr. MeTable.

[7] Lattuada G., Ragogna F., Perseghin G. (2011). Why does NAFLD predict type 2 diabetes?. Curr. Diab. Rep..

[8] Lautt W.W., Wang H.H. (2014). Obesity as an early symptom of the AMIS syndrome. J. Clin. Med..

[9] Sadri P., Reid M.A., Afonso R.A., Schafer J., Legare D.J., Macedo P.M., Lautt W.W. (2006). Meal-induced insulin sensitization in conscious and anaesthetized rat models comparing liquid mixed meal with glucose and sucrose. Br. J. Nutr..

[10] Chang C.L., Lin Y., Bartolome A.P., Chen Y.C., Chiu S.C., Yang W.C. (2013). Herbal therapies for type 2 diabetes mellitus: Chemistry, biology, and potential application of selected plants and compounds. Evid. Based Complement. Alternat. Med..

[11] Lautt W.W. (1999). The HISS story overview: A novel hepatic neurohumoral regulation of peripheral insulin sensitivity in health and diabetes. Can. J. Physiol. Pharmacol..

[12] Fernandes A.B., Patarrao R.S., Videira P.A., Macedo M.P. (2011). Understanding postprandial glucose clearance by peripheral organs: The role of the hepatic parasympathetic system. J. Neuroendocrinol..

[13] Lautt W.W., Macedo M.P., Sadri P., Takayama S., Duarte Ramos F., Legare D.J. (2001). Hepatic parasympathetic (HISS) control of insulin sensitivity determined by feeding and fasting. Am. J. Physiol. Gastrointest. Liver Physiol..

[14] Patarrao R.S., Lautt W.W., Afonso R.A., Ribeiro R.T., Guarino M.P., Fernandes A.B., Boavida J.M., Macedo M.P. (2008). Meal-induced insulin sensitization and its parasympathetic regulation in humans. Can. J. Physiol. Pharmacol..

[15] Lautt W.W., Ming Z., Legare D.J. (2010). Attenuation of age- and sucrose-induced insulin resistance and syndrome X by a synergistic antioxidant cocktail: The AMIS syndrome and HISS hypothesis. Can. J. Physiol. Pharmacol..

[16] Wang H.H., Chowdhury K.K., Lautt W.W. (2015). A synergistic, balanced antioxidant cocktail, protects aging rats from insulin resistance and absence of meal-induced insulin sensitization (AMIS) syndrome. Molecules.

[17] Ming Z., Lautt W.W. (2011). HISS, not insulin, causes vasodilation in response to administered insulin. J. Appl. Physiol..

[18] Lautt W.W., Ming Z., Macedo M.P., Legare D.J. (2008). HISS-dependent insulin resistance (HDIR) in aged rats is associated with adiposity, progresses to syndrome X, and is attenuated by a unique antioxidant cocktail. Exp. Gerontol..

[19] Ming Z., Legare D.J., Lautt W.W. (2011). Absence of meal-induced insulin sensitization (AMIS) in aging rats is associated with cardiac dysfunction that is protected by antioxidants. J. Appl. Physiol..

[20] Ming Z., Legare D.J., Lautt W.W. (2009). Obesity, syndrome X, and diabetes: The role of HISS-dependent insulin resistance altered by sucrose, an antioxidant cocktail, and age. Can. J. Physiol. Pharmacol..

[21] Ribeiro R.T., Lautt W.W., Legare D.J., Macedo M.P. (2005). Insulin resistance induced by sucrose feeding in rats is due to an impairment of the hepatic parasympathetic nerves. Diabetologia.

[22] Afonso R.A., Lautt W.W., Schafer J., Legare D.J., Oliveira A.G., Macedo M.P. (2010). High-fat diet results in postprandial insulin resistance that involves parasympathetic dysfunction. Br. J. Nutr..

[23] Chowdhury K.K., Legare D.J., Lautt W.W. (2011). Insulin sensitization by voluntary exercise in aging rats is mediated through hepatic insulin sensitizing substance (HISS). Exp. Gerontol..

[24] Chowdhury K.K., Legare D.J., Lautt W.W. (2013). Exercise enhancement of hepatic insulin-sensitising substance-mediated glucose uptake in diet-induced prediabetic rats. Br. J. Nutr..

[25] Chowdhury K.K., Legare D.J., Lautt W.W. (2013). Interaction of antioxidants and exercise on insulin sensitivity in healthy and prediabetic rats. Can. J. Physiol. Pharmacol..

[26] Chowdhury K.K., Legare D.J., Lautt W.W. (2013). Lifestyle impact on meal-induced insulin sensitization in health and prediabetes: A focus on diet, antioxidants, and exercise interventions. Can. J. Physiol. Pharmacol..

[27] Dirkx E., van Eys G.J., Schwenk R.W., Steinbusch L.K., Hoebers N., Coumans W.A., Peters T., Janssen B.J., Brans B., Vogg A.T. (2014). Protein kinase-D1 overexpression prevents lipid-induced cardiac insulin resistance. J. Mol. Cell. Cardiol..

[28] Kok B.P., Brindley D.N. (2012). Myocardial fatty acid metabolism and lipotoxicity in the setting of insulin resistance. Heart Fail. Clin..

[29] An J., Muoio D.M., Shiota M., Fujimoto Y., Cline G.W., Shulman G.I., Koves T.R., Stevens R., Millington D., Newgard C.B. (2004). Hepatic expression of malonyl-CoA decarboxylase reverses muscle, liver and whole-animal insulin resistance. Nat. Med..

[30] Hwang J.H., Stein D.T., Barzilai N., Cui M.H., Tonelli J., Kishore P., Hawkins M. (2007). Increased intrahepatic triglyceride is associated with peripheral insulin resistance: *In vivo* MR imaging and spectroscopy studies. Am. J. Physiol. Endocrinol. MeTable.

[31] Perseghin G. (2010). The role of non-alcoholic fatty liver disease in cardiovascular disease. Dig. Dis..

[32] Marchesini G., Brizi M., Morselli-Labate A.M., Bianchi G., Bugianesi E., McCullough A.J., Forlani G., Melchionda N. (1999). Association of nonalcoholic fatty liver disease with insulin resistance. Am. J. Med..

[33] Ribeiro R.T., Afonso R.A., Guarino M.P., Macedo M.P. (2008). Loss of postprandial insulin sensitization during aging. J. Gerontol. A Biol. Sci. Med. Sci..

[34] Lautt W.W., Wang X., Sadri P., Legare D.J., Macedo M.P. (1998). Rapid insulin sensitivity test (RIST). Can. J. Physiol. Pharmacol..

[35] Lautt W.W. (2003). Practice and principles of pharmacodynamic determination of HISS-dependent and HISS-independent insulin action: Methods to quantitate mechanisms of insulin resistance. Med. Res. Rev..

[36] Snyder F., Stephens N. (1959). A simplified spectrophotometric determination of ester groups in lipids. Biochim. Biophys. Acta.

[37] Tardi P.G., Mukherjee J.J., Choy P.C. (1992). The quantitation of long-chain acyl-CoA in mammalian tissue. Lipids.

[38] Poanta L.I., Albu A., Fodor D. (2011). Association between fatty liver disease and carotid atherosclerosis in patients with uncomplicated type 2 diabetes mellitus. Med. Ultrason..

[39] Guarino M.P., Afonso R.A., Raimundo N., Raposo J.F., Macedo M.P. (2003). Hepatic glutathione and nitric oxide are critical for hepatic insulin-sensitizing substance action. Am. J. Physiol. Gastrointest. Liver Physiol..

[40] Lautt W.W., Schafer J., Macedo M.P., Legare D.J. (2011). Bethanechol and *N*-acetylcysteine mimic feeding signals and reverse insulin resistance in fasted and sucrose-induced diabetic rats. Can. J. Physiol. Pharmacol..

[41] Lautt W.W. (2004). A new paradigm for diabetes and obesity: The hepatic insulin sensitizing substance (HISS) hypothesis. J. Pharmacol. Sci..

